# Genome-wide analysis of long noncoding RNA and mRNA co-expression profile in intrahepatic cholangiocarcinoma tissue by RNA sequencing

**DOI:** 10.18632/oncotarget.15721

**Published:** 2017-02-24

**Authors:** Wenhui Yang, Yuan Li, Xia Song, Jun Xu, Jun Xie

**Affiliations:** ^1^ Department of Biochemistry and Molecular Biology, Shanxi Medical University, Taiyuan, Shanxi, China; ^2^ Shanxi Province Cancer Hospital, Affiliated Cancer Hospital of Shanxi Medical University, Taiyuan, Shanxi, China; ^3^ Department of Thoracic Surgery, National Cancer Center/Cancer Hospital, Chinese Academy of Medical Sciences and Peking Union Medical College, Beijing, China

**Keywords:** intrahepatic cholangiocarcinoma, long noncoding RNA, co-expression, RNA sequencing

## Abstract

Long noncoding RNAs (lncRNAs), which are pervasively transcribed in the genome, are emerging in molecular biology as crucial regulators of cancer. RNA-seq data were downloaded from GEO of NCBI and further analyzed to identify novel targets in intrahepatic cholangiocarcinoma (iCCA). We investigated differences in lncRNA and mRNA profiles between 7 pairs of iCCA and adjacent normal tissues. 230 lncRNAs were differentially expressed more than four-fold change in iCCA tissues. Among these, 97 were upregulated and 133 downregulated relatively to normal tissues. Moreover, 169 lncRNAs and 597 mRNAs formed the lncRNA-mRNA co-expression network which consist 766 network nodes and 769 connection edges. Bioinformatics analysis identified these dysregulated lncRNAs were associated with cholesterol homeostasis, insoluble fraction and lipid binding activity and were enriched in complement and coagulation cascades and PPAR signaling pathway. These results uncovered the landscape of iCCA-associated lncRNAs and co-expression network, providing insightful information about dysregulated lncRNAs in iCCA.

## INTRODUCTION

Cholangiocarcinoma (CCA) is the most common biliary malignancy and the second most common hepatic malignancy after hepatocellular carcinoma [1]. Intrahepatic cholangiocarcinomas (iCCAs) are hepatobiliary cancers with features of cholangiocyte differentiation, which are located within the hepatic parenchyma [2]. iCCA is an aggressive malignancy with 5-year survival rate of less than 10% [2]. Surgery is the only curative option for iCCA. However, the resectability rate is low because patients typically present at advanced stages where there is no accepted standard of care [3, 4]. Therefore, the study of iCCA remains extremely important to improve the detection or therapy of iCCA.

In past decades, multiple important signaling pathways in tumorigenesis had been uncovered by analyzing the expression profiling of coding genes. etc. To the updated knowledge, actively transcribed long noncoding RNAs (lncRNAs) identified by high-throughput platform are involved in even more complexed cancer genome regulatory networks. LncRNAs are endogenous cellular RNA transcripts longer than 200 nucleotides in length and without protein coding capacity [5]. LncRNAs, generally expressed at a lower level than coding genes, are emerging as crucial regulators of cancer in molecular biology and display more tissue-specific and cell-specific expression patterns [5–7].

LncRNAs are poorly conserved and have been shown to control every level of multi-level regulated gene expression pathway via cis or trans-acting mechanism [8]. Therefore, analysis of the co-expression of lncRNAs and mRNA can help to predict their functional role in the development of various diseases, including cancer and lay a foundation for uncovering the mechanism ultimately [9]. Dysregulated lncRNAs have been identified in breast cancer [10, 11], lung cancer [12, 13], colorectal cancer [14], renal cell carcinoma [15], and hepatocellular carcinoma [16–18], etc., indicating that certain lncRNAs may participate in tumorigenesis. However, few lncRNA involved in iCCA has been revealed and understanding of lncRNAs in tumor biology is still in infancy especially in iCCA.

In this study, we reported profiles of differentially expressed lncRNAs and mRNAs in 7 pairs of iCCA and adjacent normal tissues. In particular, we evaluated the mRNAs that are co-expressed with the differentially expressed lncRNAs during the genesis of iCCA.

## RESULTS

### Differentially expressed lncRNAs and mRNAs in iCCA tissues

**Figure 1:**
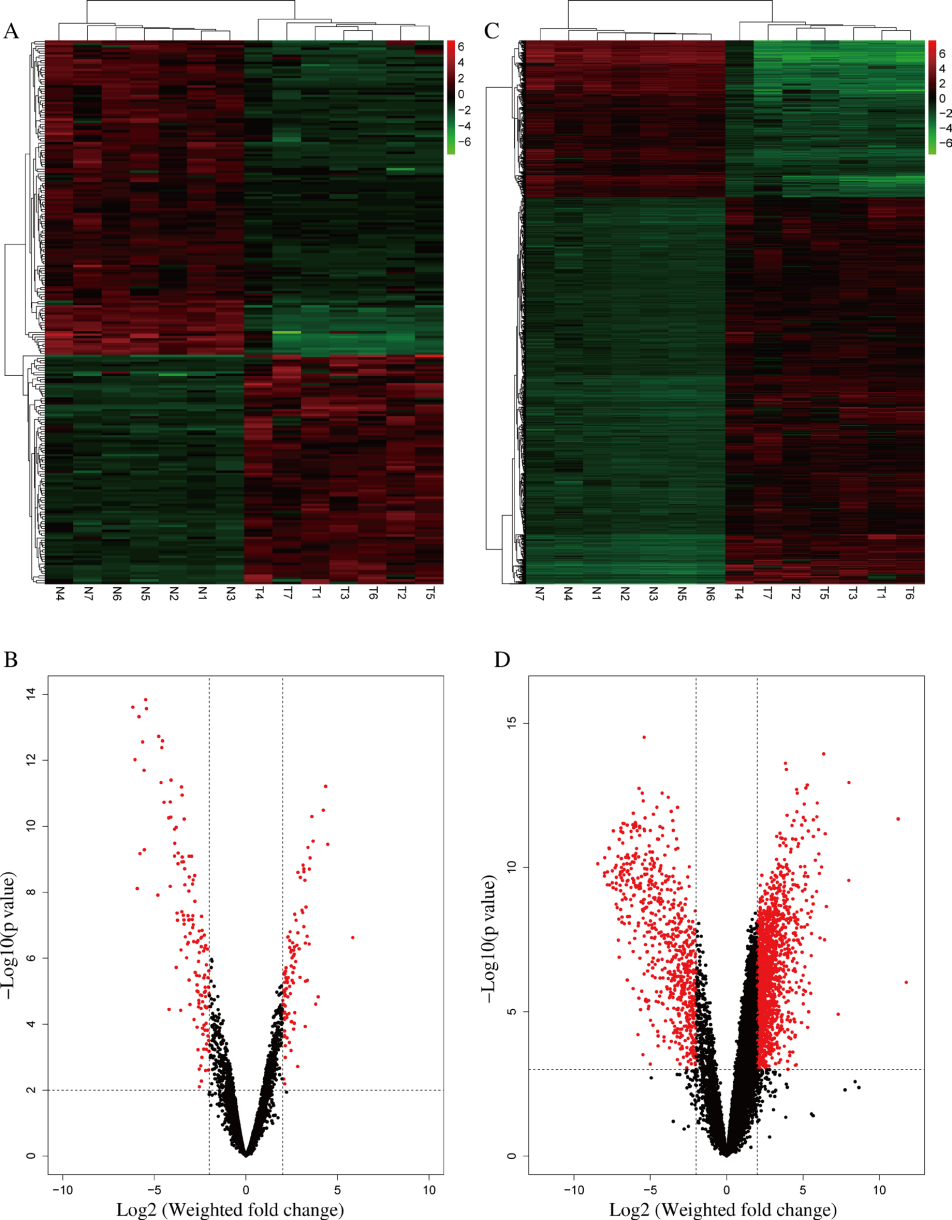
Differentially expressed lncRNAs and mRNAs in iCCA (**A**) Heatmap of expression profiles for the 230 lncRNAs that showed significant expression changes (133 down-regulated and 97 up-regulated), red through green color indicates high to low expression level. (**B**) Volcano plot of the *P* values as a function of weighted fold-change for lncRNAs in the 7 normal and 7 tumor tissues. Dark dots represent lncRNAs not significantly differentially expressed (fold change <4, *P* > 0.01) and red dots represent lncRNAs significantly differentially expressed (fold change ≥ 4, *P* < 0.01). (**C**) Heatmap of expression profiles for 2220 mRNAs that showed significant expression changes (640 down-regulated and 1580 up-regulated), red through green color indicates high to low expression level. (**D**) Volcano plot of the *P* values as a function of weighted fold-change for mRNAs in the 7 normal and 7 tumor tissues. Dark dots represent mRNAs not significantly differentially expressed (fold change <4, *P* > 0.001) and red dots represent lncRNAs significantly differentially expressed (fold change ≥ 4, *P* < 0.001).

From the RNA-seq data, a comparison of lncRNA expression profile between the 7 pairs of iCCA and adjacent normal tissues identified 230 lncRNAs that were differentially expressed (fold change ≥ 4, *P* < 0.01) between iCCA and the normal tissues (Figure 1A–1B, Supplementary Table 1). Among them, 97 lncRNAs were upregulated and 133 lncRNAs were downregulated (Figure 1A–1B, Supplementary Table 1). RP11-328K4.1, LINC01093, LINC00844, RP11-372E1.4 and ITIH4-AS1 were the five most significantly down-regulated lncRNAs while RP11-532F12.5, AC016735.1, RP11-284F21.7, LINC01123 and AC013275.2 were the five most significantly up-regulated lncRNAs in iCCA, respectively (Table 1).

**Table 1:** The five most significantly down- and up- regulated lncRNAs with their predicted targets

Ensemble ID	Gene Symbol	Regulation	Log_2_FC	*P* value	Targets*
ENSG00000248740	RP11-328K4.1	Down	5.36	7.90E–13	TP53/TGFA/AP2B1
ENSG00000249173	LINC01093	Down	6.34	7.90E–13	PPP2R1B/PRKX/C3
ENSG00000237949	LINC00844	Down	6.01	9.24E–13	APOE/PLG/CIDEB
ENSG00000243818	RP11-372E1.4	Down	5.18	1.43E–12	ADH1C/CLEC4M
ENSG00000239799	ITIH4-AS1	Down	5.10	1.99E–12	C19orf80/NDRG3/MVP
ENSG00000261183	RP11-532F12.5	Up	4.35	1.76E–09	*NA*
ENSG00000224739	AC016735.1	Up	4.23	7.33E–09	*NA*
ENSG00000229953	RP11-284F21.7	Up	3.60	1.10E–08	ATP1B1/FITM1
ENSG00000204588	LINC01123	Up	3.67	4.90E–08	EFEMP1/ZNF431/TUBB3
ENSG00000231013	AC013275.2	Up	4.47	5.73E–08	IRAK1/JRKL/RHOC

*mRNA targets for the lncRNAs were identified by Spearman correlation test (Coef > 0.95, *P* < 0.001), only three targets were listed if there were more and *NA* represented no target was identified under the above conditions.

Meanwhile, mRNA expression profiles in iCCA were compared with the noncancerous tissues. 2220 mRNAs were found differentially expressed (fold change ≥ 4, *P* < 0.001) between iCCA and the noncancerous tissues. Among them, 640 mRNAs were downregulated and 1580 mRNAs were upregulated (Figure 1C–1D, Supplementary Table 2).

Then we asked whether these transcripts of 230 lncRNAs and 2220 mRNAs could distinguish iCCA from normal tissues. Figure 1A and 1C showed that the 7 iCCA samples are clustered together in one group and clearly separated from the samples of normal tissue in both heatmaps. The overall changes from a respective normal to cancer state were also seen separately as a difference in expression profile of either the lncRNA or the mRNA (Figure 1A, 1C). These observations suggest that a potential dynamic interaction between lncRNAs and mRNAs may be reshaping the landscape of the whole transcriptome during iCCA development.

### Significantly co-expressed mRNA in iCCA tissues

**Figure 2:**
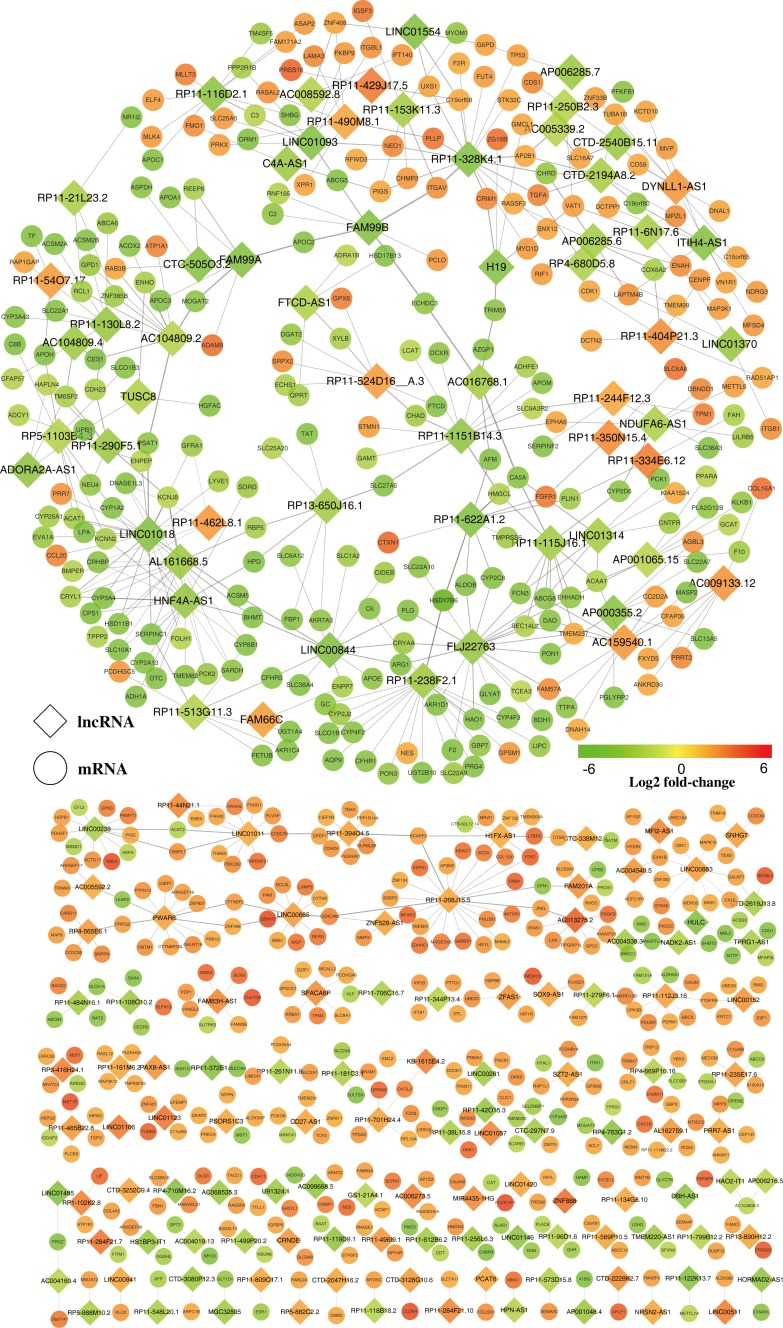
Predicted lncRNA and mRNA co-expression network in iCCA The co-expression network was established between the 169 significantly expressed lncRNAs and 597 significantly differentially expressed mRNAs that had Spearman correlation coefficients equal to or greater than 0.95. Within this co-expression network, 550 pairs presented as positive, and 219 pairs presented as negative. The diamonds represent lncRNAs while the circles represent mRNAs. Red through green color indicates high to low expression level.

Genome-wide gene expression profiling of both lncRNAs and mRNAs from iCCA and normal tissue was conducted to detect possible associations of lncRNAs with iCCA. We predicted the potential target mRNAs for the 230 differentially expressed lncRNAs by using the Spearman's correlation tests. As results, 597 mRNAs (Coef > 0.95, *P* < 0.001) were targeted by 169 lncRNAs (61 lncRNAs had none targets) (Table 1, Figure 2, Supplementary Table 3). Among them, 219 mRNAs were negatively correlated and 550 mRNAs were positively correlated (Figure 2, Supplementary Table 3).

### Construction of the co-expression network

We constructed a co-expression network of these dysregulated lncRNAs and their targeted mRNAs. Differently expressed lncRNAs and their significantly correlated mRNAs were imported to draw the network using Cytoscape (version 3.2.1). The co-expression network was composed of 766 network nodes and 769 connection edges between 169 lncRNAs and 597 mRNAs (Figure 2). Within this co-expression network, 550 pairs presented as positive, and 219 pairs presented as negative (Supplementary Table 3). Strikingly, over one third (59 in 169) of the lncRNAs and their correlated mRNAs were integrated in one complex network by sharing the same mRNAs (Figure 2). Moreover, this co-expression network revealed that one lncRNA could target up to 22 mRNAs and one coding gene could correlate with up to four lncRNAs (Figure 2, Supplementary Table 3).

### Go and KEGG pathway analysis

**Figure 3:**
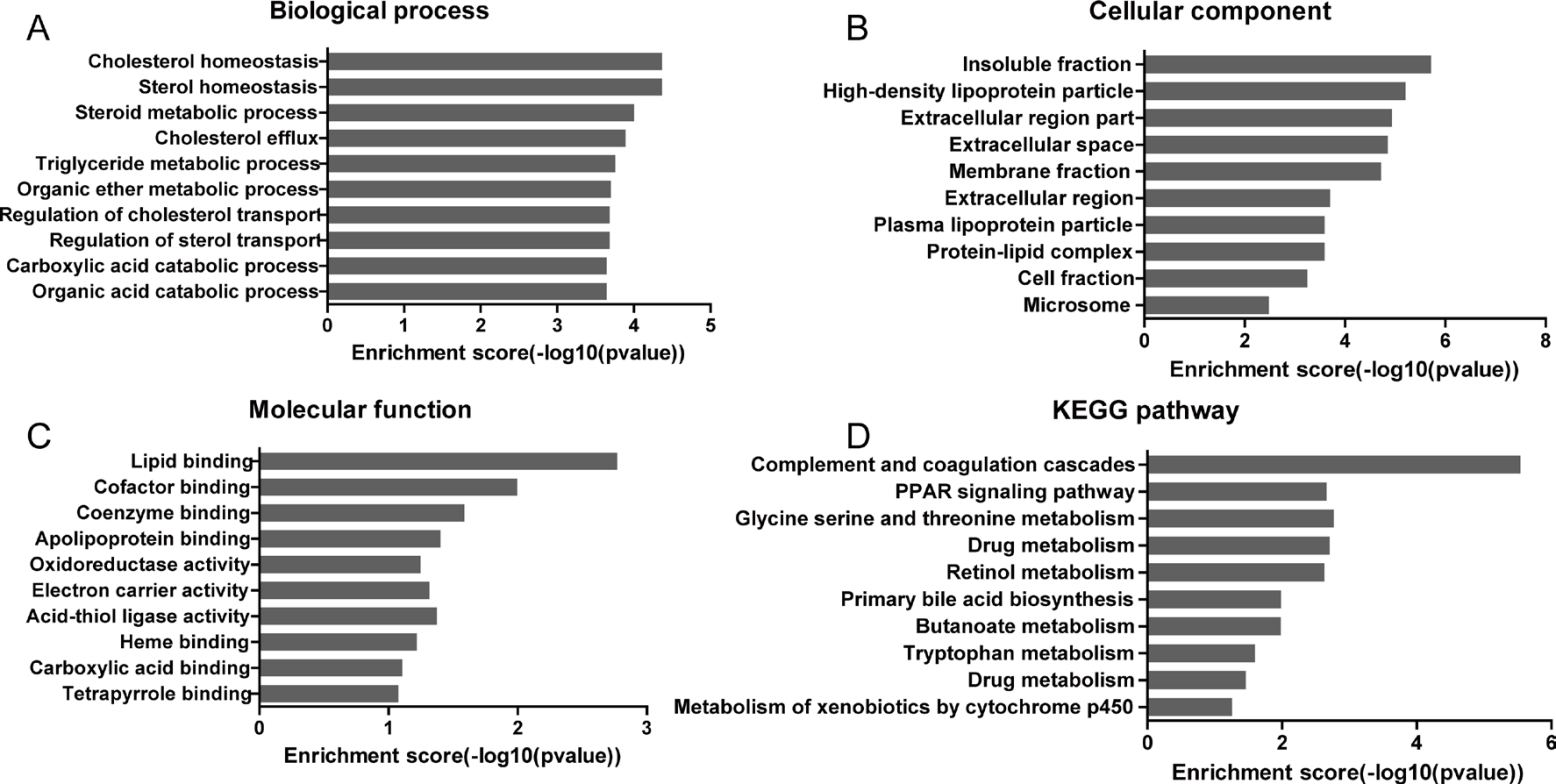
GO and KEGG analysis of the significantly correlated mRNAs targeted by lncRNAs The ontology covers three domains: (**A**) Biological Process, (**B**) Cellular Component and (**C**) Molecular Function. (**D**) KEGG pathway enrichment analysis of the significantly correlated mRNAs targeted by lncRNAs.

A GO enrichment analysis was applied to explore the functions of co-expressed mRNAs identified in this study. Genes were organized into hierarchical categories to uncover gene regulatory networks on the basis of biological process, cellular component and molecular function. Specifically, two-side Fisher's exact test was used to determine the GO category and GO annotation list was greater than expected by chance (*P* value < 0.05 is recommended as the cut-off). Through GO analysis we found that these dysregulated transcripts of lncRNAs were associated with cholesterol homeostasis and sterol homeostasis (ontology: biological process), insoluble fraction and high-density lipoprotein particle (ontology: cellular component), lipid binding and cofactor binding activity (ontology: molecular function). The genes corresponding to the mRNAs 448 genes involved in biological processes, 443 genes involved in cellular components and 429 genes involved in molecular functions (Figures 2, 3A–3C, Supplementary Table 4).

Significant pathways of co-expressed mRNAs were compared with the KEGG database to further specify and identify target mRNAs among the 597 identified genes. Through the pathway analysis, we identified that 9 pathways were significantly enriched among the transcripts (Figure 3D, Supplementary Table 5). Among them, complement and coagulation cascades (hsa04610), PPAR signaling pathway (hsa03320) and glycine serine and threonine metabolism (hsa00260) were the 3 significantly enriched networks respectively with FDR correction (FDR < 0.05). Some of these pathways, such as the classical gene category ‘drug metabolism’ and ‘metabolism of xenobiotics by cytochrome P450’ signaling pathway, have been reported to be involved in drug resistance in cancer, but the enrichments were not significant after FDR correction (Figure 3D, Supplementary Table 5).

## DISCUSSION

During the past two decades of molecular biological studies on human iCCA, a number of coding genes have been identified genetically or epigenetically responsible for iCCA development. A few reports have studied the function of certain coding genes or genetic and epigenetic alterations in pathogenesis of human CCA [2]. However, these studies have produced no definitive results, because they analyzed a limited number of genes in CCA, without separate analysis of different subtypes [2].

Moreover, to date, a small but growing number of lncRNAs have been experimentally investigated [19] and a view is emerging that lncRNAs can serve as signal, decoy, guide or scaffold molecules in regulation of gene expression [5]. Thus, this study was conducted to investigate the role of lncRNA in iCCA genesis. However, unlike microRNAs, increasing evidence has confirmed that lncRNAs not easily predicted based on lncRNA sequence [20, 21]. Thus, predicting potential cancer-related lncRNAs by integrating various kinds of biological data is one of the most important and attracting topics for biology research.

LncRNA H19 and HULC have been reported to play important roles in other tumors. Current evidence indicates that H19 plays crucial roles in tumor metastasis, through the regulation of critical events specifically the epithelial to mesenchymal and the mesenchymal to epithelial transitions [22]. HULC promotes tumor angiogenesis in liver cancer through miR-107/E2F1/SPHK1 signaling [23] and modulates abnormal lipid metabolism in hepatoma cells through a miR-9-mediated RXRA signaling pathway [24]. We compared our results with a microarray study which reported dysregulated lncRNA profile in iCCA [25]. Though the role of H19 in tumor initiation and progression has long been a subject of controversy and HULC is highly over expressed in serval tumors, H19 and HULC were significantly downregulated in iCCA tissues compared with normal tissues in both studies. Meanwhile, LINC01559, GS1-600G8.5 and FAM99A, LINC00844 were also significantly upregulated or downregulated respectively in both studies. Moreover, lncRNA UCA1 contributes to progression of hepatocellular carcinoma through inhibition of miR-216b and activation of FGFR1/ERK signaling pathway [26] and is upregulated in breast cancer [27], colorectal cancer [28], esophageal squamous cell carcinoma [29] and bladder carcinoma [30]. We detected a similar expression pattern of lncRNA UCA1 in our study. Thus, at least the above aberrant lncRNAs may be linked to iCCA tumorigenesis.

To date, few studied has reported dysregulation of lncRNAs in iCCA tissues and our data is the first RNA-seq analysis revealing 230 lncRNAs aberrantly expressed in iCCA tissues with fold changes of four or more. For instance, RP11-328K4.1 was the most significantly down-regulated lncRNAs in iCCA compared to the normal tissue and was significantly correlated with TP53, TGFA and AP2B1 (Table 1), which were key regulators in many tumors. Next, 169 the differentially expressed lncRNAs and 597 target mRNAs were integrated into one co-expression network. Bioinformatics analysis revealed that these dysregulated lncRNAs were associated with cholesterol homeostasis (ontology: biological process), insoluble fraction (ontology: cellular component) and lipid binding (ontology: molecular function), and were enriched in 9 gene pathways, e.g., complement and coagulation cascades and PPAR signaling pathway. Complement and coagulation cascades are implicated in many physiological and pathological processes including the inflammatory processes, which are important contributing factors to tumorigenesis once dysregulated [31]. Besides, PPARs are linked to metabolic disorders and are interesting pharmaceutical targets in cancer [32, 33]. PPARs seem to have contradictory roles in tumorigenesis serving as an oncogene or tumor suppressor which might be related to different isoforms of PPARs. Recent studies indicate that PPARα, which iscommonly expressed in many tumor cell lines [34–36], could suppress colon carcinogenesis tumor development [37] and inhibit melanoma cell metastasis [38]. On the contrary, PPARγ ligands have been shown to promote differentiation and apoptosis in a variety of cancer cells including colon cancer [39], prostate cancer, gastric cancer [40], bladder cancer [41], breast cancer [42]. Mounting evidences suggest that PPARs involve in drug sensitivity and PPARs agonists have antitumor effects *in vitro* and *in vivo* in several cancers. Recent research reported that PPAR ligands were shown to upregulate the expression of human organic cation transporter type 1(hOCT1), leading to an increase in imatinib (the gold standard for the treatment of chronic myeloid leukemia) uptake [43], resulted in inhibiting cell growth and inducing differentiation and apoptosis. However, there were other studies suggest that PPARs agonists may cause some tumors. Several dual PPAR agonists induce bladder tumor and sarcomas formation in rodents [44, 45]. PPARβ/δ were implicated in the development of colon cancer [46], also stimulated the cell line proliferation of human breast and prostate cancers [47].

Do PPARs ligands suppress or promote the development of CCA? There were evidence suggesting anti-tumor effects of PPARs in CCA cell lines. PPARγ was expressed in CCA cell lines, its ligand inhibited the cell growth by inducing apoptosis and by cell cycle regulation (G1 arrest), aslo by p53-dependent mechanisms [48, 49]. In addition, increasing researches has proved that epithelial-to-mesenchymal transition (EMT) as a mechanism promoting dissemination in CCA [50]. Some studies have demonstrated that activation of PPARγ could inhibit TGF-β-induced EMT and prevents metastasis by antagonizing Smad3 function [51]. PPARγ also could increase Spry4 expression by Wnt7A/Fzd9 signaling then induced a reversal of the epithelial to mesenchymal transition [52]. Taken together, these results suggest that PPARs could inhibit or reversing EMT in CCA.

These results were specific in liver biological process and were significantly associated with clinical information. The present study demonstrated that lncRNAs might participate in the tumorigenesis of iCCA. The iCCA is a fatal bile duct cancer with dismal prognosis and limited therapeutic options, and its etiology and molecular pathogenesis remain largely unknown. Further studies will be needed to conclusively demonstrate and elucidate the precise role of lncRNAs in iCCA. This proof-of-principle study provides potential lncRNA targets for further investigations on molecular pathogenesis of iCCA.

## MATERIALS AND METHODS

### Data curation and reprocessing

Transcriptome sequencing data was downloaded from public available Gene Expression Omnibus (GEO, http://www.ncbi.nlm.nih.gov/geo/) under the accession number GSE63420 [53]. In brief, raw RNA-seq reads were aligned and mapped by TopHat v2.0.9 and transcriptome assemblies were performed by Cufflinks v2.1.1 with the default parameters [54, 55]. Only expressed genes were considered and the threshold of the expression value was set to 0.001. In this study, human lncRNA and protein-coding gene annotation was directly downloaded from GENECODE v22. All of the categories in the “long non-coding RNA gene annotation” GTF file were considered lncRNAs. To obtain genome-wide lncRNA and protein-coding gene expression profiles, normalized expression data were subsequently analyzed for differently expressed lncRNAs and mRNAs using Bioconductor packages (limma, version 3.26.1) [56] in R (version 3.2.2) with default parameters. Differentially expressed lncRNAs and mRNAs were identified through fold change filtering.

### Visualization of the lncRNA-mRNA regulatory network

The Spearman's correlation test was used to estimate the co-expression relationships between the lncRNAs and mRNAs. Moreover, the significance *P*-value of the correlation coefficient was estimated. Finally, a set of co-expression genes of each lncRNA were identified under coefficient threshold of 0.95 and significance threshold of 0.001. “Guilty by association” is employed and the filtered co-expressed genes were defined as the potential targets of the lncRNAs in this study. Using Cytoscape (version 3.2.1), associations between lncRNAs and mRNAs were connected by solid lines to build the lncRNA-mRNA co-expression network.

### Bioinformatics analysis

Gene Ontology (GO) analysis is a functional analysis associating differentially expressed mRNAs with GO categories. The predicted target genes above were input into the Database for Annotation, Visualization and Integrated Discovery (DAVID; http://david.abcc.ncifcrf.gov/), which utilized GO to identify the molecular function represented in the gene profile. Furthermore, we also used the KEGG (Kyoto Encyclopedia of Genes and Genomes) database (http://www.genome.ad.jp/kegg/) to analyze the potential functions of these target genes in the pathways.

### Statistical analyses

The expression levels of lncRNAs and mRNAs that were differentially expressed between iCCA and normal tissues were compared using Bioconductor package (limma version 3.26.1) and R (version 3.2.2) software. Co-expression relationships between the lncRNAs and mRNAs were estimated by Spearman correlation test. The false discovery rate (FDR) was also calculated to correct the *P* value and statistical significance was considered as *P* < 0.05 unless stated.

## SUPPLEMENTARY MATERIALS TABLES












