# Supplementary material for: Genome-wide analysis of long noncoding RNA and mRNA co-expression profile in intrahepatic cholangiocarcinoma tissue by RNA sequencing
Source: Oncotarget. 2017 Feb 24;8(16):26591–9. doi: 10.18632/oncotarget.15721 (PMC5432281; doi:10.18632/oncotarget.15721)
Supplement: Supplementary file 1 [file oncotarget-08-26591-s001.pdf]

Genome-wide analysis of long noncoding RNA and mRNA co-expression profile in intrahepatic cholangiocarcinoma tissue by RNA sequencing

Supplementary Materials

Supplementary Table 1: Signicantly differentially expressed lncRNAs in iCCA.
See Supplementary_Table_1

Supplementary Table 2: Significantly differentially expressed mRNAs in iCCA.
See Supplementary_Table_2

Supplementary Table 3: Significantly correlated lncRNAs and mRNAs.
See Supplementary_Table_3

Supplementary Table 4: GO analysis. See Supplementary_Table_4

Supplementary Table 5: KEGG pathway enrichment. See Supplementary_Table_5
